# Synergistic effects of agonistic costimulatory antibodies adsorbed to amphiphilic poly(γ-glutamic acid) nanoparticles

**DOI:** 10.1186/2051-1426-1-S1-P128

**Published:** 2013-11-07

**Authors:** Catja Freiburghaus, Sissela Broos, Linda C Sandin, Thomas H Tötterman, Takami Akagi, Mitsuru Akashi, Carl Borrebaeck, Peter Ellmark, Malin Lindstedt

**Affiliations:** 1 Department of Immunotechnology, Lund University, Lund, Sweden; 2 Alligator Bioscience AB, Lund, Sweden; 3 Department of Immunology, Genetics and Pathology, Uppsala University, Uppsala, Sweden; 4 Department of Applied Chemistry, Graduate School of Engineering, Osaka University, Osaka, Japan

## 

Agonistic antibodies targeting costimulatory pathways for the immune system hold great potential for cancer immunotherapy. However, systemic administration of immunoactivating antibodies can be associated with side effects such as cytokine release syndrome and liver toxicity. Polymeric nanoparticles (NPs) represent an exciting approach to control the release of therapeutic antibodies and to optimize the desired immune response via selective targeting. With increased potency as objective, agonistic antibodies targeting TNF receptors were adsorbed to immune stimulating biodegradable and self-assembled polymeric nanoparticles composed of γ-glutamic acid (γ-PGA NPs). The effects of antibodies targeting CD40, CD137 and OX40 were evaluated based on T and B cell proliferation as well as on cytokine release and phenotypic maturation of antigen-presenting cells. Our results demonstrated a strong synergistic effect on human B cell proliferation of CD40 monoclonal antibodies (mAbs) carrying NPs in vitro. In addition adsorption of anti-CD40 mAb to γ-PGA NPs significantly reduced the systemic release of TNF-α, IL-6, IL-10 and IL-12, compared to treatment with the soluble mAb. Preliminary results indicate increased T cell proliferation and activation by CD137 and OX40 agonistic antibodies in combination with γ-PGA NPs. Combining NPs with agonistic antibodies for cancer immunotherapy offers intriguing opportunities for increased therapeutic efficacy and safety.

